# Public perception of xenotransplantation and its implication for policy and regulation: lessons learnt from a qualitative study of the Swedish public

**DOI:** 10.3389/ti.2026.16483

**Published:** 2026-09-03

**Authors:** Rui Liu, Markus Idvall, Susanne Lundin

**Affiliations:** 1 Department of Arts and Cultural Sciences, Lund University, Lund, Sweden; 2 Department of Ethnology, History of Religions and Gender Studies, Stockholm University, Stockholm, Sweden

**Keywords:** policy implication, public opinion, qualitative analysis, questionnaire survey, xenotransplantation, public engagement

## Abstract

The field of xenotransplantation is advances rapidly. Its cross-species nature creates regulatory challenges different from those in allotransplantation. Public opinion is promoted as an essential element in developing responsible governance for this technology. This study takes a qualitative thematic approach to analyze data collected through a questionnaire (n = 136) to the Swedish public. The aim is, first, to analyze how respondents articulate concerns, hopes, and moral evaluations regarding XT, and second, to reflect on what these responses reveal about the methodological conditions required for meaningful public engagement in the development of regulatory frameworks for emerging biomedical technologies. The findings demonstrate that public views are not directly associated with the technical assessment of risk and benefit but are anchored in shifting sociocultural perceptions regarding human survival, animal welfare, and vulnerability as individuals move between different scenarios. We conclude that developing policy and regulations for XT require continuous engagement with the underlying public opinions. We learned that meaningful public engagement with emerging biomedical technologies involves sustained processes of familiarization, communication, and dialogue between science, society, and policy.

## Graphical Abstract

**Figure:**
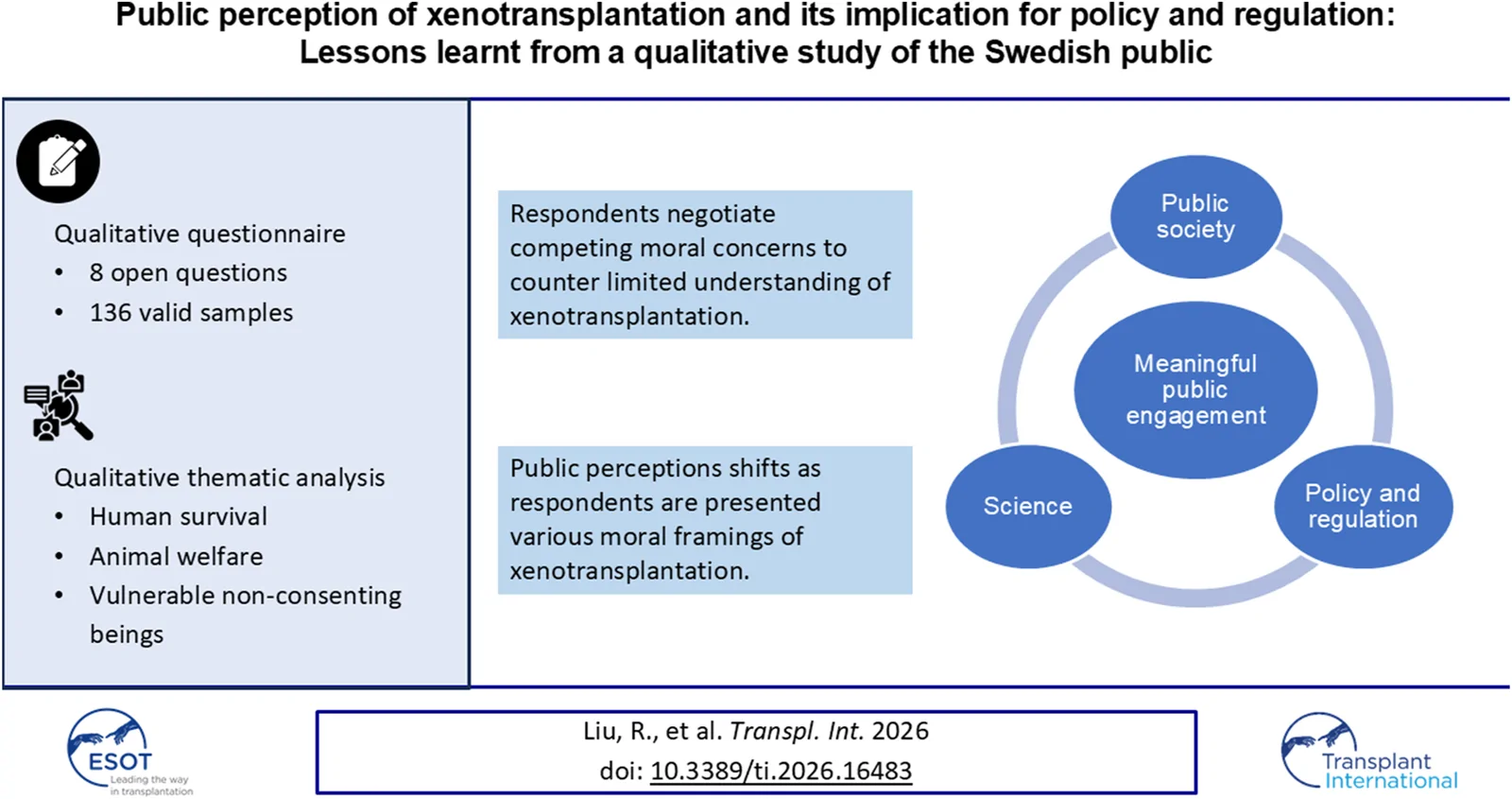

## Introduction

The field of xenotransplantation (XT) is advancing rapidly, marked by the world’s first transplant of a transgenic pig heart to a living human in 2022, pig kidney transplant in 2023, and the launch of clinical trials involving pig kidneys in the United States in 2025. This technology offers hopes for alleviating the critical shortage of human donor organs. However, its cross-species nature introduces profound uncertainties including unknown viral risks, shifting human-animal relations, and the rise of a bioeconomy around engineered organs [1]. These complexities require regulatory frameworks capable not only of assessing technical safety but also of addressing broader societal concerns.

Public involvement is increasingly recognized as essential for legitimate governance of technology. The World Health Organization explicitly endorses a democratic approach, stating that regulatory systems overseeing XT should be transparent, include scientific and ethical assessment, and involve the public [2]. This endorsement is in line with research into responsible innovation and anticipatory governance, arguing for broader public engagement in the early development stage of emerging technologies [3]. Public engagement in this regard is not only a means of identifying acceptance or resistance but a way of clarifying societal concerns, strengthening democratic legitimacy, and informing regulatory development [4, 5].

However, involving the public in the governance of XT presents significant challenges. Earlier studies by the authors, conducted as part of a multidisciplinary cross-country EU project (2008–2012) examining citizen participation in XT development, show that relevant knowledge was primarily concentrated among medical scientists and civil servants, who were also the main participants in policy development [6]. This illustrates a democratic challenge: although public participation is formally endorsed, the knowledge required to participate meaningfully may be unevenly distributed. Recent empirical studies likewise suggest that XT remains unfamiliar to many members of the public [7]. Public understandings of the technology may also be shaped by the process through which the technology is introduced, explained, and discussed [8]. This suggests that public opinions should not be treated as pre-existent or stable but as emerging through the very process of public involvement itself. This has methodological implications and an impact on how questions are posed and analyzed.

Existing studies on public views of XT primarily rely on quantitative approaches, measuring the levels of acceptance, perceived risks, and willingness to receive animal organs [7, 9]. While these studies identified divergent views from different groups of the public, their results provided limited insights into how individuals interpret potential risks and hope and on how public opinions may become ambiguous when they engage with the subject.

Our study responds to these gaps by exploring how members of the Swedish public perceive and make sense of the risks, hopes, and moral implications associated with XT. The study was designed to examine how respondents mobilize moral and cultural framings when encountering XT as an emerging biomedical technology. During analysis, however, the material also revealed the instability and ambiguity of public sense-making depending on the different scenarios considered. This prompted us to treat the material not simply as evidence of public perceptions of XT but as an opportunity to reflect on methodological appropriateness in the context of emerging and relatively unfamiliar technologies.

The aim of this article is therefore twofold. First, we analyze how respondents articulate concerns, hopes, and moral evaluations regarding XT. Second, we reflect on what these responses reveal about the methodological conditions required for meaningful public engagement in the development of regulatory frameworks for emerging biomedical technologies.

In the remaining text, we describe the research design, data collection, sample characteristics, and analytical approach. We then present findings showing how respondents’ perceptions of XT shifted across different scenarios. The article ends with a reflexive discussion of the methodological challenges of engaging the public in the democratic governance of emerging biomedical technologies.

## Materials and methods

In this article, our analysis draws primarily on the qualitative data collected in a two-stage research design. A population-based quantitative survey among the Swedish public was conducted in February 2025, which collected 1038 valid samples. The results indicate dividing views and a high level of hesitancy among the Swedish public concerning whether the society should invest in developing XT and whether one would consider participating in a xenograft clinical trial. Detailed analyses were published in a peer-reviewed research letter [10]. The findings of the survey data were used to inform the second stage of the research, which was a qualitative open-ended questionnaire among the Swedish public. The questionnaire was distributed in May 2025 through Lund University’s Folklife Archive digital panel and the authors’ research network.

### Data collection

Respondents were provided with information about the research project and how their data will be used. Those who consented proceeded to a short introduction text (see the English translation in Appendix A) about the recent development of XT and its potential to alleviate organ shortages. Demographic information, including gender, age, residential regions, and levels of education, was collected. Eight open-ended questions were included, where respondents wrote their answers without word limits. Table 1 listed the questions translated to English by the authors.

**TABLE 1:** Eight open-ended questions in the questionnaire.

Q1	Have you heard of “xenotransplantation” before? On what occasions or through which channels? What thoughts, feelings, or images came to mind in connection with this term?
Q2	Xenotransplantation is considered a promising way to address the shortage of human organs. However, such transplantations may involve risks. For example, viruses may spread between species. Do you think researchers should continue to develop xenotransplantation if safety can be ensured? Why or why not?
Q3	Do you think Sweden will become a suitable place for xenotransplantation internationally? Please describe your reasoning
Q4	If xenotransplantation becomes a common medical practice in the future, do you think the technology could reduce or reinforce equal access to organs, both in Sweden and globally? Please elaborate
Q5	Private companies are investing heavily in xenotransplantation technology. Do you think the public health sector or government authorities should lead the development? Why or why not?
Q6	What would you think about receiving a pig organ if this were the only option for survival for you or your family? Please describe what you would think about the advantages and disadvantages of xenotransplantation for yourself or your family
Q7	At some stage, it may be conceivable that clinical trials on humans will be necessary for xenotransplantation to fully function as a medical treatment. Would you be willing to participate in such trials? What is your opinion on this?
Q8	Xenotransplantation means that animals must be genetically modified to be compatible with humans. The technology has raised different views on ethical issues concerning animal welfare. What is your opinion on this?

Q1 sought to screen respondents’ familiarity with XT and how they relate to the technology. Q2-Q8 introduced selected dimensions of XT to elicit respondents’ moral evaluations of its risks and benefits and their views on governance, health inequality, and animal welfare. Our intention was to invite respondents to reason about XT through a series of scenarios and issues that are prominent in ethical and regulatory debates.

In total, 136 valid samples were collected and included in the analysis. Most respondents self-identified as females (68.4%), lived in cities (68.4%), and had post-secondary education longer than 3 years (69.1%). The mean age was 51.9 years, with respondents ranging from 20 to 86 years old. Table 2 presents a descriptive analysis of the respondents’ demographic background.

**TABLE 2:** Respondents’ demographic background.

Characteristic	Category	n	%
Gender	Female	93	68.4
	Male	39	28.7
	Non-binary	2	1.5
	Prefer not to say	1	0.7
	Missing	1	0.7
Age group	18–29	19	14.0
	30–44	36	26.5
	45–64	37	27.2
	65+	44	32.4
Residential area	Urban	93	68.4
	Smaller town	25	18.4
	Rural area	16	11.8
	Missing	2	1.5
Education level	Elementary school or equivalent	1	0.7
	Upper secondary school or equivalent	12	8.8
	Post-secondary education, shorter than 3 years	29	21.3
	Post-secondary education, longer than 3 years	94	69.1

### Data analysis

The open-ended responses were analyzed using a qualitative thematic approach [11]. Given the questionnaire format and the relatively brief nature of many responses, the analysis did not aim to reconstruct comprehensive personal narratives. Instead, we focused on identifying recurring patterns through which respondents interpreted, evaluated, and morally negotiated risks and hopes brought about by XT across the full dataset.

All responses were first read repeatedly by all authors to gain familiarity with the material. The first stage of coding was inductive and focused on identifying recurring issues, expressions, and forms of reasoning in the responses. Initial codes captured, for example, references to organ shortage, survival, safety, viral risk, animal welfare, genetic modification, public versus private responsibility, inequality, consent, vulnerability, and trust in Swedish healthcare and regulation. Coding was conducted across the full dataset of responses rather than question by question, allowing us to identify how similar or diverging opinions appeared in relation to different scenarios.

In the second stage, related codes were grouped into broader themes. This process involved comparing responses across questions and examining how respondents connected different ethical and social concerns. For example, the human-animal hierarchy appeared both in responses concerning personal or family survival and in responses concerning animal welfare, while concerns about vulnerability and consent appeared in relation to both human experimentation and the use of genetically modified animals. The themes were therefore not treated as mutually exclusive categories or as fixed respondent positions. Rather, they represent recurring and sometimes overlapping, even self-contradictory, moral frameworks through which respondents made sense of XT.

The preliminary themes were then reviewed and refined through discussion among the authors. During this process, we considered whether the themes were sufficiently supported by the material, whether they captured variation and ambiguity in respondents’ answers, and how they related to ethical and sociocultural debates on XT. The final themes were selected because they captured central tensions in the material: the prioritization of human survival, the moral status and welfare of animals, and issues of vulnerability of non-consenting beings. Table 3 presents the initial codes and categories, subthemes, and themes.

**TABLE 3:** Themes, subthemes, and codes/categories identified in the material.

Main themes	Subthemes	Categories/codes
Human survival	Immediate rejection of animal organs	Disgust; refusal; “madness”; ethical rejection of breeding or killing animals for human survival
	Ambivalence under survival pressure	Survival instinct; uncertainty; family or loved ones; species hierarchy; conflict between ethical principles and a desire to live
	Reframing death and survival	Acceptance of mortality; questioning survival as overriding priority; critique of technological extension of life
Animal welfare	Normalization through existing animal use	Meat industry comparison; animal use already accepted; genetic modification in food production; XT as not morally exceptional
	Conditional acceptance based on welfare	Avoiding unnecessary suffering; minimizing harm; welfare thresholds; sacrifice justified by human benefit
	Human-animal hierarchy	Human life assigned more moral weight; animal suffering weighed against human benefit; species hierarchy
Vulnerability of non-consenting beings	Animals’ inability to consent	Lack of animal agency; inability to refuse; moral standing of animals; human domination over animals
	Moral discomfort among supporters	Support combined with unease; consent as a “stumbling block”; unresolved ethical burden
	Responsibility for children and loved ones	Parental responsibility; decision-making for vulnerable others; reconsidering opposition when children are involved

The analysis was a reflexive process. As mentioned earlier, the study was designed with the assumption that respondents would be able to reflect on XT after receiving introductory information through the questionnaire. During the analysis, however, it became clear that many responses drew on general sociocultural and ethical reasoning rather than detailed knowledge of the technology. Therefore, apart from analyzing the collected data, we also looked at the research design and considered in which way the question formulations may have influenced respondents’ perceptions of XT.

### Ethical approval

This project was approved by the Swedish Ethical Review Authority (DNR 2024-04088-01). All quotes presented in the next section were translated by the authors from Swedish.

## Results

When responses were analyzed question by question, they often appeared to fall into positions of support for or opposition to XT. However, when comparing responses from the same individuals across different scenarios, these positions were less stable. Many respondents oscillated between support, hesitation, and opposition depending on the provided scenarios. These shifts drew our attention to the instability and ambiguity of public perceptions. In the following, we present the ambivalence around three central issues: human survival, animal welfare, and the vulnerability of non-consenting beings.

### Human survival

The idea of using animal organs to prolong human life invoked a range of reactions from strong opposition to ambivalent acceptance. Some rejected the scenario by answering a single word with exclamation marks such as “Madness!” (survey response 24), “No!!!!!!” (survey response 27), and “Disgusting!” (survey response 30). Others used a more moderate tone, for example, “I think it is unethical that another life should be bred and raised only to die for my sake.” (survey response 39). Some were more hesitant when survival was at stake:

I would not feel comfortable with this. If it is the only option for survival, it is currently difficult for me to know how I would act. Ideally, I would see other solutions. (survey response 42).

Furthermore, some respondents explicitly claimed that they were firmly against XT but could also admit that, like one respondent phrased it, “there is clearly a very strong desire for survival for loved ones or me.” (survey response 38) The ethical dilemma puts some individuals in a morally conflicted situation, where existential pressure challenged their ethical principles. “I still hope”, the aforementioned respondent continued,

that I would have said no because I do not believe that we can build a good world if we are simultaneously exploiting, killing, and experimenting on other fellow beings in various ways. I think I would have been ashamed of myself if I had said yes to an organ from another animal that was killed so that I could get its organ! (survey response 38).

The above response illustrates how the survival scenario could unsettle a respondent’s moral position. Accepting an animal organ was imagined as a source of shame, while refusing it might feel difficult because of the desire to stay alive. Rather than taking a stable stance, the respondent articulated a tension between ethical opposition to animal exploitation and the desire to live. When survival became an immediate urgency, choices were made under conditions of pressure and uncertainty, raising questions about how regulatory frameworks can protect individuals from being positioned as solely responsible for ethically difficult questions.

While many considered the technology as posing a moral dilemma, several respondents raised the issue of how human beings face death and the short span of individual life expectancy in the long human history. One person wrote.

Death is something everyone should learn to face with less fear. [We should] spend money on making people feel better instead and not be so afraid of everything. I would not accept an organ from an animal and would not pressure anyone close to me to do so either. (survey response 49).

The response above does not simply reject XT. It also questions the premise that survival should always be treated as the overriding ethical priority. By reframing death as something to be accepted rather than technologically deferred, the respondent challenges the survival-oriented logic that often underpins medical innovation.

### Animal welfare

Like the survival scenario, animal welfare elicited both moral unease and a ‘but-then’ attitude among respondents. Despite acknowledging the ethical challenges and potential harm to animals, those in favor of XT justified their opinions in different ways. Some brought up the ever-expanding meat industry, suggesting that XT would not change the situation for animal use in human society that much:

It is difficult to know whether the genetic modifications have a negative impact on animals when their organs are to be used on humans. This is certainly a smaller impact than the genetic modification that has been carried out to increase, for example, meat production. (survey response 89).

The comparison between consuming pigs as food and as medical products normalizes the use of animals in human life. It situates the technology within an already existing and largely routinized practice of genetic modification. XT in this way is framed as neither extraordinary nor morally disruptive. This rhetoric framing also shifts the moral burden away from potential recipients to those who develop, regulate, and implement the technology. The responses imply that XT development concerning animal use would need to be addressed at the level of governance and regulation. This was commented on by another respondent:

Clearly it is a shame for the animals, but if you look around the meat industry, there are already many animals for whom it is not so fun. (survey response 40).

Some supporters defended their position by arguing that the value of humans is greater than that of animals. For instance, one person wrote.

I think a human is ‘more important’ than an animal and that it is therefore justified to conduct animal experiments that cause the animal pain and death. What I hope for in the future is that we learn to grow these organs from created beings that lack consciousness. That is, we learn to grow the organs. I don’t know how reasonable this is, but I maintain that causing suffering to a few animals is justified if the purpose is to benefit humans. (survey response 16).

In the above quote, animal suffering was not unproblematic. Echoing many other responses, it was disturbing. Nevertheless, it was not considered a violation or unethical act *per se* if human lives could be saved.

In sum, these quotes suggest conditional acceptance of XT on a gradient. The supportive stance is grounded both in a value system where humans are assigned more moral weight than animals and in the normalized meat consumption to sustain human life. Yet, while some insisted that animal welfare should not be worsened, others perceived that sacrifice was needed to develop the technology. This was exemplified in a comment: “As long as the animals are not subjected to unnecessary suffering, any restrictions on the welfare of the animals are a sacrifice that can be made.” (survey response 44).

### Vulnerability of non-consenting beings

The vulnerability of non-consenting beings emerged as another main diverging point among the respondents. Several respondents stated that animals cannot consent to XT and therefore should not be subjected to biomedical manipulation. Their argument critiques the power of humans over animals and emphasizes animals’ inability to make their own decisions or express refusal. One respondent believed that pigs are “fantastically intelligent animals” but added “[H]ow can they say no?” (Survey response 86). Such responses framed consent not merely as a procedural requirement but as a marker of moral agency and moral standing. Hence, consent, or the lack of it, becomes a way for respondents to draw and question boundaries of agency, vulnerability, and responsibility between humans and animals.

Those in favor also raised the issue that animals cannot consent, however, their concern seemed less a decisive ethical consideration and more a sense of moral discomfort. Several respondents expressed that the absence of animal consent was troubling. One individual described the animal aspect as “a difficult stumbling block”, and asked rhetorically “What right does man have to dominate animals that cannot give their consent?” (survey response 107). Another person similarly noted that the practice was “an abuse of the animals, since they have no capacity to make their own decisions to contribute.” (survey response 111).

When a different figure of a non-consenting being, namely the human child, was involved, the scenario shifted respondents’ moral stances. It revealed a dilemma between taking parental responsibility and holding one’s ethical principles. Grounded in the tension is moral responsibility to protect a vulnerable child. Those in favor then place a little extra emphasis on the potential benefit for children. One respondent, for example, felt positive about XT but not for herself, explaining “if it concerned the lives of my children or grandchildren, I would gladly accept help.” (survey response 88).

Among respondents who were opposed to XT in other scenarios, the ethical dilemma involving children appeared to shake their opinions. Some became more refrained in their criticism or even open to change their position. For example, one respondent initially stated that “we must stop using animals for everything, their lives are no less valuable than ours”. Nevertheless, she acknowledged the difficulty of maintaining this principle if her own children were affected:

If someone had offered me an organ from an animal to save my child, I don’t know if I could say no either, that doesn’t mean it’s right. It’s better that that alternative doesn’t exist.” (Survey response 101).

This response captured a recurring form of ambivalence in the material. The responsibility to save a child makes refusal to XT less straightforward. In this regard, the scenario does not produce a stable for or against position. Rather, it reveals how public perceptions shift when abstract ethical principles are brought into contact with concrete situations involving responsibility and vulnerability.

## Discussion

This study set out to analyze how members of the Swedish public articulate concerns, hopes, and moral evaluations regarding XT and to reflect on what these responses reveal about the methodological conditions required for meaningful public engagement in the development of regulatory frameworks for emerging biomedical technologies. The findings show that public perceptions of XT are scenario-dependent. For example, respondents who rejected the technology on personal ethical grounds changed their views when imagining the survival of their children or loved ones. Those feeling positive about the technology in the first place became hesitant when considering animals’ inability to give consent. Furthermore, prevalent meat consumption and culturally embedded ideas of human hierarchy helped some respondents justify XT development, while the same vocabulary framed in other scenarios, particularly those around suffering, genetic manipulation, and vulnerability, prompted them to question XT.

As we have shown, many respondents drew on overlapping moral repertoires: the value of human life, discomfort with animal suffering, established practices of animal use, responsibility for children and family members, and concern for non-consenting beings. Yet, these repertoires result in inconsistent opinions even from the same individuals. Taken together, the findings suggest public views are ambivalent forms of sense-making that emerge as individuals encounter, engage, and reason through the various moral framings of XT.

This has important methodological implications. The study was designed to explore public perceptions of XT through open-ended questions after respondents had received introductory information about the technology. However, as we went through the material, it revealed that many respondents reasoned through broad ethical intuitions and familiar moral comparisons rather than specific understanding of XT. In retrospect, this is a limitation of our research design. Nevertheless, it reflects a broader methodological challenge in studying public engagement with emerging technologies. When a technology such as XT is unfamiliar and not yet embedded in everyday experience, public views may not pre-exist the research encounter in a stable form. They may instead take shape through the way the technology is introduced, explained, and imagined in particular scenarios.

This reflexive insight sheds light on democratic governance and the regulation of emerging technologies. Public engagement is not a one-off measurement of attitudes. Ambiguous or shifting responses are not to be simply interpreted as a lack of knowledge. The ambivalence, as we have shown, indicates that respondents are actively negotiating competing moral concerns under conditions of limited familiarity with XT. To meaningfully engage the public, therefore, requires processes that enable public familiarization, contextual understanding, and deliberation about the conditions under which the technology should or should not be developed [12]. An implication for research design is that researchers should not assume respondents’ prior knowledge about XT. Policy-wise, public engagement is not merely used to anticipate resistance or communicate expert-defined benefits and risks. Rather, spaces need to be created where society can learn about the technology, question its purposes, and deliberate on its ethical and social consequences [13].

The study has several limitations. The qualitative material was generated through open-ended questionnaire responses rather than interviews, focus groups, or deliberative forums. Many responses were brief, which limited the possibility of reconstructing detailed personal narratives or deeply situated cultural interpretations. The questionnaire also introduced XT through selected scenarios, including organ shortage, risk, survival, clinical trials, inequality, public-private investment, and animal welfare. These framings may have shaped the issues respondents considered. In addition, the sample included a relatively high proportion of females, urban residents, and respondents with higher education. These limitations mean that the findings should not be interpreted as representative of the Swedish public. Nevertheless, the material provides insight into how respondents begin to reason about XT when invited to engage with it as an unfamiliar and ethically complex biomedical technology.

In conclusion, this study shows that public perceptions of XT are not best understood as fixed positions for or against the technology. Rather, respondents mobilized shifting and sometimes conflicting moral framings when considering XT across different scenarios. We also learned throughout the study a broader methodological and democratic challenge: meaningful public engagement with emerging biomedical technologies requires sustained processes of familiarization, communication, and dialogue between science, society, and policy.

## Data Availability

The datasets presented in this article are not readily available to comply with the Swedish Ethical Review Authority. Requests to access the datasets should be directed to the corresponding author.

## Appendix A Information for potential questionnaire respondents

*English translation by the authors:* The use of cells, tissues, and heart valves from animals has long been an established technique in transplant medicine. For example, pigs are often used to replace human heart valves. Researchers have now begun to explore the possibility of transplanting whole pig organs into humans. Developments in this field are progressing rapidly. In 2022, the world’s first pig-to-human heart transplant was performed. In 2025, the US Food and Drug Administration approved a clinical trial involving American patients. Xenotransplantation is considered a promising technique for addressing the global shortage of human organ donations. However, many uncertainties must be addressed in parallel with the development of the technology.

This questionnaire explores public opinion on the implications of xenotransplantation.
